# Effectiveness of Different Irrigant Activation Systems on Smear Layer Removal: A Scanning Electron Microscopic Study

**DOI:** 10.3390/jcm11041003

**Published:** 2022-02-15

**Authors:** Ramón Miguéns-Vila, Benjamín Martín-Biedma, Saleta Aboy-Pazos, David Uroz-Torres, Pablo Álvarez-Nóvoa, Ana Belén Dablanca-Blanco, Iván Varela-Aneiros, Pablo Castelo-Baz

**Affiliations:** 1 Máster Endore, University of Santiago de Compostela, 15705 Santiago de Compostela, Spain; ramon.miguens.vila@gmail.com (R.M.-V.); saleta.aboy@gmail.com (S.A.-P.); pablo.alvarez.novoa@gmail.com (P.Á.-N.); dablanca.91@gmail.com (A.B.D.-B.); ivan.varela.aneiros@gmail.com (I.V.-A.); pablocastelobaz@gmail.com (P.C.-B.); 2 Faculty of Dentistry, University of Granada, 18071 Granada, Spain; daviduroz@endodontico.com

**Keywords:** EndoVac, irrigant activation, Irrisafe, passive ultrasonic irrigation, ProUltra PiezoFlow, scanning electron microscopy, smear layer

## Abstract

The aim of this study was to evaluate the effectiveness of smear layer removal after the use of different irrigation methods (passive ultrasonic irrigation (PUI), continuous ultrasonic irrigation (CUI), apical negative pressure irrigation and conventional irrigation) using scanning electron microscopy (SEM) as an analytical tool. A total of 100 single-canal teeth were decoronated and randomly divided into five groups (*n* = 20) according to the irrigation method used: conventional irrigation with front outlet syringe, conventional irrigation with lateral outlet syringe, apical negative pressure irrigation (EndoVac), PUI with Irrisafe and CUI with ProUltra PiezoFlow ultrasonic irrigation needle. Root canal preparation was performed with the ProTaper Gold system up to the F4 instrument, and 5.25% NaOCl was used as an irrigant. After chemical-mechanical preparation, the roots were split longitudinally, and the coronal, middle and apical thirds were examined. SEM digital photomicrographs were taken at ×1000 magnification to evaluate the amount of smear layer in each root canal third; CUI significantly removed more smear layer than any other irrigant activation protocol (*p* < 0.05); CUI was more effective in removing the smear layer than the other irrigation protocols. However, none of the irrigation protocols were able to produce root canals completely free from smear layer.

## 1. Introduction

Smear layer can be defined as an irregular layer of organic and inorganic components, formed on the dentinal walls during root canal instrumentation [1]. It acts as a barrier to the penetration of irrigants and sealers into the dentinal tubules, therefore hindering proper disinfection and sealing of the root canals [1,2]. Although sodium hypochlorite (NaOCl) has been recommended as the main irrigant solution during canal preparation [3,4], it does not remove the inorganic component of the smear layer [4,5]. Thus, its association with a chelating agent, such as ethylenediaminetetraacetic acid (EDTA), is more than necessary to provide an effective removal of this layer from the root canal walls [4,5,6,7].

Due to the complexity of the root canal system, especially in the apical third with its ramifications and lateral canals and their intrinsic inaccessibility to the mechanical action of instruments, the disinfection procedure relies on an effective delivery of irrigating solutions [8,9]. The use of irrigants can efficiently remove the smear layer which is the key for successful root canal treatment [4,10]. Although conventional irrigation with syringe has been the most used technique to irrigate root canals, the replenishment and exchange of the irrigant are limited in the apical part of this system [11]. In this sense, several irrigant techniques have been proposed to overcome this limitation.

EndoVac (Discus Dental, Culver City, CA, USA), an apical negative pressure (ANP) irrigation system, was designed to safely deliver irrigants to apical areas and unreachable parts of the root canal system through a negative pressure mechanism, extruding less irrigant to the periapical area and decreasing the risk of NaOCl accidents [12,13]. Another commonly used technique to irrigate root canals is the application of ultrasonically activated irrigation, which can either be intermittent or continuous [4,14,15,16]. While intermittent passive ultrasonic irrigation (PUI) requires a vibrating file or tip within the canal and replenishment of the solution with a syringe after each activation cycle, the continuous ultrasonic irrigation (CUI) method is delivered through an ultrasonically activated irrigation needle, not requiring irrigant replacement between each ultrasonic file activation.

Based on this background, the aim of this study was to evaluate, through scanning electron microscopy (SEM), the efficacy of PUI, CUI and ANP irrigant activation techniques in the removal of smear layer from the root canal walls. Conventional irrigation with a front outlet (CIFO) and a side-vented (CISV) syringe was used as a reference technique for comparison.

The null hypothesis was that no difference would be found among the activation protocols in smear layer removal.

## 2. Materials and Methods

### 2.1. Ethics Declarations

All biological samples were included after obtaining informed consent from all subjects. This study was carried out in accordance with relevant guidelines and regulations, followed after ethics committee approval (Comité de Ética de Investigación de Galicia).

### 2.2. Sample Selection and Preparation

After the approval of local ethics committee, a total of 100 single-canal straight maxillary human teeth, extracted for periodontal reasons, were stored in 2% thymol solution until their use. The specimens were decoronated to obtain a standardized root length of 15 mm using a diamond disc (Komet Dental, Lemgo, Germany) and a surgical handpiece (Kavo Dental, Biberach an der Riss, Germany). The external surface of each root, including the apex, was sealed with nail polish to prevent the extrusion of irrigants through the apical foramen, after the placement of a size 10 K-file (Dentsply Sirona Endodontics, Ballaigues, Switzerland) at the working length (WL) to prevent the nail polish from entering the canal [4].

### 2.3. Root Canal Instrumentation

The specimens were fixed in the Pro-Train device (Simit Dental, Mantova, Italy) to allow the operator to perform the root canal instrumentation procedures. The WL was determined electronically using a size 10 K-file connected to the Root ZX apex locator (Morita, Osaka, Japan), which was confirmed with radiographs (Ultra-speed E; Kodak, Rochester, NY, USA). A glide path was performed with the PathFile system (Dentsply Sirona Endodontics) using the 0.13, 0.16 and 0.19 instruments. Then, the sample was prepared using the ProTaper Gold system (Dentsply Sirona Endodontics) following the sequence recommended by the manufacturer: S1, S2, F1, F2, F3 and F4 instruments at a speed of 250 rpm and 5.2 Ncm maximum torque. During instrumentation, each root canal was irrigated with 5.25% NaOCl (Parcan; Septodont, Saint-Maur-des-Fosses, France) using a side-vented needle (Max-I-Probe; Hawe Neos Dental SA, Bioggio, Switzerland) by inserting it into the canal as far as possible. Final irrigation was performed with 3 mL of 5.25% NaOCl, 1 mL of 17% EDTA (Coltene Whaledent, Langenau, Germany) for 1 min [17] followed by 3 mL of 5.25% NaOCl [18,19].

### 2.4. Final Irrigant Protocols

After root canal preparation, the sample was randomly allocated into five groups (*n* = 20), according to the final irrigant protocol used:

**PUI group.** A 200 µm ultrasonic file (Irrisafe; Satelec, Bordeaux, France) driven by the P5 Newtron ultrasonic system (Acteon; Mount Laurel, NJ, USA), at a power setting of 5, was placed at 1 mm short of the WL and activated in 1–2 mm up-and-down movements with 5.25% NaOCl for 30 s, followed by 17% EDTA for 30 s and 5.25% NaOCl for 30 s. Irrigation was performed at a flow rate of 15 mL/min per specimen.

**CUI group.** A P500 µm ultrasonic irrigation needle (ProUltra PiezoFlow; Dentsply Sirona Endodontics) connected to the P5 Newtron ultrasonic system, at a power setting of 5, was positioned 1 mm short of binding, no deeper than 2/3 of the WL, and activated in 1–2 mm up-and-down movements with 5.25% NaOCl for 30 s, followed by 17% EDTA for 30 s and 5.25% NaOCl for 30 s. Irrigation was performed at a flow rate of 15 mL/min per specimen.

**ANP group.** Firstly, macroirrigation was performed with 5.25% NaOCl for 30 s using the macrocannula. Then, 3 cycles of microirrigation were performed. For the first cycle, the microcannula was inserted 1 mm short of the WL, and 5.25% NaOCl was continuously restocked for 20 s. The two successors’ microirrigation cycles were similar but used 17% EDTA and, lastly, 5.25% NaOCl.

**CIFO group.** A conventional syringe irrigation was performed with a front-outlet NaviTip needle (Ultradent Products Inc.; South Jordan, UT, USA) positioned at 1 mm short of the WL using 5.25% NaOCl for 30 s followed by 17% EDTA for 30 s and 5.25% NaOCl for 30 s.

**CISV group.** The same sequence of the CIFO group was used herein. The only difference was the use of a side-vented needle (Max-I-Probe).

A total of 20 mL of irrigant was used per canal in all groups, with a flow rate of 15 mL/min with the same irrigation and activation time in each group. After the final irrigant protocols, the root canals were dried with absorbent paper points (Dentsply Sirona Endodontics). The same experienced operator performed all chemical-mechanical procedures.

### 2.5. SEM Preparation and Evaluation

After the final irrigant protocols, the specimens were grooved at 4, 8 and 12 mm from the root apex, defining the coronal, middle and apical thirds, respectively. The most coronal 3 mm of each root was discarded. Then, SEM was used to evaluate the smear layer on each root surface. Longitudinal grooves were made on the buccal and lingual surfaces of each root with a diamond disc, without penetrating the root canal, to facilitate its posterior fracture with a chisel. An F4 gutta-percha master cone (Dentsply Sirona Endodontics) was placed inside the root canal to prevent contamination during root fracture. Only the half of each root that conserved the most visible part of its root canal was used; the other half was discarded.

For sample fixation, the protocol described by Perdigão et al. [20] was used. Briefly, the specimens were immersed in 2.5% glutaraldehyde/2% paraformaldehyde in 0.1 M sodium cacodylate buffer at pH 7.4 for 12 h at 4 °C. Then, they were rinsed with 10 mL 0.1 M sodium cacodylate buffer, pH 7.4, for 2 h and postfixed with 2% osmium tetroxide in 0.1 M sodium cacodylate buffer for 1 h, followed by washing in 0.1 M sodium cacodylate for 1 h. The specimens were rinsed four times with deionized water and dehydrated through ascending grades of ethanol. After sample fixation and dehydration with ethanol, each specimen was orificated for observation under SEM (FE-SEM; Sigma, Carl Zeiss, Jena, Germany; secondary electron emission mode, 5 KV accelerated voltage). Microphotographs were taken at ×1000 magnification to evaluate the presence of smear layer at the root canal surface in the center of each root third.

Two independent examiners, trained in the scoring process and with concordance verified with the κ test, scored the samples following the criteria described by Torabinejad et al. [21]: 0 = no smear layer (absence of smear layer on the surface of the root canal, all dentinal tubules clean and open), 1 = moderate smear layer (no smear layer on the surface of the root canal, but dentinal tubules contain debris) and 2 = heavy smear layer (smear layer covers the root canal surface and dentinal tubules). The final results of the smear layer removal analysis were obtained by statistical analysis of the scores for each root third in each of the five experimental groups.

### 2.6. Statistical Analysis

Significant differences in the amount of smear layer removal achieved by the final irrigant protocols were sought using non-parametric Pearson’s χ^2^ test. The level of statistical significance was set at *p* < 0.05. Distribution of the variables was not normal (Shapiro–Wilk test *p* < 0.05), and they were not homogenous (Levene test *p* < 0.05). All statistical analyses were performed using the SPSS software (ver. 20.0; SPSS Inc., Chicago, IL, USA).

PASS 15.0 software (NCSS, LLC., Kaysville, UT, USA) was used for sample size and power of the test calculation. The minimum size for each group was determined to be 4 to achieve a power of 0.9 and a significance level of 0.05.

## 3. Results

CUI significantly removed more smear layer than any other irrigant activation protocol (*p* < 0.05), with 43.1% of the specimens in this group showing no smear layer. PUI removed significantly more smear layer than ANP, CIFO and CISV (*p* < 0.05). No significant difference was found among the other groups (*p* > 0.05). The results are summarized in Table 1.

**Figure 1:**
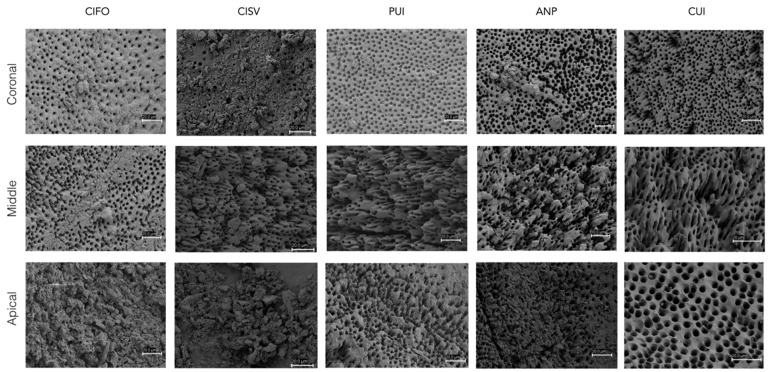
Representative SEM images of the coronal, middle and apical root thirds of each irrigation protocol (×1000 magnification, secondary electron mode, 5 KV accelerated voltage).

Significant differences among the root thirds were observed in all experimental groups (*p* < 0.05), with the middle third showing a more pronounced removal of smear layer (Table 1), meaning that complete removal of smear layer with any system was higher in the middle third than the other two. CUI and PUI significantly removed more smear layer than the other groups in both middle and apical thirds (*p* < 0.05), while, in ANP and CISV groups, none of the samples showed a complete absence of smear layer in the apical third. Figure 1 shows representative images of all root thirds of the five experimental groups. The apical image of the CUI system shows an example of a sample with no smear layer, whereas the apical part of the CIFO images exhibits an exemplification of smear layer in dentinal tubules and the surface. A middle amount of smear layer is shown on the apical image of the PUI system (Figure 1).

## 4. Discussion

The null hypothesis was rejected as differences were found among the activation protocols in smear layer removal (alternative hypothesis).

Overall, the present results show that CUI removed significantly more smear layer than any other final irrigation protocol (*p* < 0.05). This result is in agreement with the findings of Bueno et al. [22], which also show that CUI performed better than PUI and conventional irrigation in the removal of smear layer from the root canal walls of mandibular pre-molars. This outcome may be explained by the greater output force of the irrigant as well as the strong cavitation generated by this approach.

Regarding the analysis by root thirds, the results reveal no difference in the smear layer removal in the coronal third, which is in line with previous studies [19,23]. The enhanced removal of smear layer in this specific root third is relative to a higher volume and accessibility of irrigants in this area as well as a greater density of dentinal tubules [24]. On the other hand, no group showed a complete removal of the smear layer in the apical third. This outcome might be explained by the smaller volume of irrigant reaching this area and the lesser density of dentinal tubules [24]. The results of this in vitro study show that both ultrasonic methods (CUI and PUI) were more effective in smear layer removal in the apical third, which is consistent with the results of Caron et al. [25] and Blank-Gonçalves et al. [26], as these authors also found that CUI achieved the highest levels of smear layer removal in the entire root canal.

The lack of performance of conventional irrigation with lateral and frontal outlet needles (CSIFO, CSILV) may be attributed to the existence of an air bubble inside the canal (known as vapor lock), which may prevent the irrigant from accessing the apical third in a proper way. On the contrary, the ultrasonic-activated protocols, such as CUI and PUI, have the ability to disintegrate the vapor lock, facilitating the delivery of irrigants to the apical region [27].

The in vitro conditions were optimized using a closed-apex model, which provides adequate conditions to reproduce the vapor lock phenomenon [28]. In addition, to avoid design errors, the volume of NaOCl and EDTA used during the final irrigation process was the same for all samples analyzed as well as the usage time of each protocol [18].

Although the removal of the smear layer is dependent on the use of a chelating substance [23], final irrigation protocols can drive the chelator into inaccessible areas, thereby improving its efficacy. In the present study, single-canal teeth were standardized to a working length of 15 mm and then instrumented with the ProTaper Gold system up to the F4 instrument. A wide apical preparation was performed, as in similar studies [29], with the objective of increasing the cleaning efficacy in the apical thirds [29,30], to provide a more effective irrigant flow in the apical region and enhance the efficacy of the irrigants [29]. It was also done to expose more dentinal tubules to the action of the irrigants [29].

Plotino et al. [29] noted that PUI was one of the systems in their study that removed significantly more smear layer from the apical third. This result is consistent with ours, as CUI and PUI were the methods that were more effective in the apical third. However, Abraham et al. [31], also with SEM evaluation, found that diode laser and sonic activation were more effective than PUI in removing smear layer from the apical part of the root canals. Those two activation methods were not used in our study; nonetheless, there were differences in the methodology as Abraham et al. [31] used 0.2% chitosan as irrigating solution.

Different anatomies such as curved root canals have more difficulties in regards to disinfection and complete removal of smear layer. This was confirmed in a SEM study by Haupt et al. [32], which found that no activation technique was able to completely eliminate debris and smear layer from root canal walls in curved canals. In our study, complete removal of smear layer was found in some of the samples due to the efficacy of some activation systems but also to the enhanced disinfection that can be achieved in straight root canals.

Smear layer and debris, as was done in other studies [29,33,34], were examined via SEM evaluation of the coronal, middle and apical third of the canals. SEM evaluation has several limitations as an analysis method. Firstly, the images of this study were quantified by a scoring system, which is a bias as it is invariably subjective. Moreover, it may be difficult to check all areas of the root canal for cleanliness; thus additional shots were taken for some samples to double-check if the canal walls were clean or not [35]. In addition, SEM smear layer removal assessments do not take into consideration the distribution of sclerotic dentin, which can cause misinterpretation in the photos [29,36].

Another potential limitation of this study is the shape of the canal as it may alter the results in a way that the wider and more oval is the canal, the more difficult it is to touch the canal walls.

The results of this in vitro research, as with other in vitro studies, need to be validated in in vivo conditions to evaluate the application of the irrigation systems in clinical situations and check if they can alter the treatment outcomes.

## 5. Conclusions

Under the conditions of this study, it can be concluded that CUI was more effective in removing the smear layer than PUI and ANP, using CSIFO and CSISV as reference. Irrigant activation systems (PUI, CUI and ANP) showed more success in removing the smear layer than the “classical” irrigation methods (CSIFO and CSICV) using SEM. However, none of the irrigation protocols were able to produce root canals completely free from smear layer.

## Figures and Tables

**Table 1:** Number of specimens and percentage regarding the smear layer scores in the root thirds of all experimental groups.

		No Smear Layer (*p* < 0.05) *	Smear Layer in Dentinal Tubules, Clear Dentinal Surface	Smear Layer in Dentinal Tubules and Surface	
CSIFO	Coronal third	4 (20%)	13 (65%)	3 (15%)	*p* = 0.038 *
	Middle third	5 (25%)	11 (55%)	4 (20%)	
	Apical third	1 (5%)	8 (40%)	11 (55%)	
CSISV	Coronal third	1 (5%)	16 (80%)	3 (15%)	*p* = 0.002 *
	Middle third	6(30%)	12 (60%)	2 (10%)	
	Apical third	0 (0%)	7 (35%)	13 (65%)	
PUI (*p* < 0.05)	Coronal third	2 (10%)	15 (75%)	3 (15%)	*p* = 0.009 *
	Middle third	10 (50%)	8 (40%)	2 (10%)	
	Apical third	3 (15%)	10 (50%)	7 (35%)	
ANP	Coronal third	4 (20%)	8 (40%)	8 (40%)	*p* = 0.017 *
	Middle third	5 (25%)	13 (65%)	2 (10%)	
	Apical third	0 (0%)	9 (45%)	11 (55%)	
CUI	Coronal third	6 (30%)	9 (45%)	5 (25%)	*p* = 0.001 *
	Middle third	16 (80%)	4 (20%)	0 (0%)	
	Apical third	9 (45%)	11 (55%)	0 (0%)	

* Statistical difference.
